# Activation of an endothelial Notch1-Jagged1 circuit induces VCAM1 expression, an effect amplified by interleukin-1β

**DOI:** 10.18632/oncotarget.6456

**Published:** 2015-12-03

**Authors:** Federica Verginelli, Laura Adesso, Isabelle Limon, Anna Alisi, Marie Gueguen, Nadia Panera, Ezio Giorda, Lavinia Raimondi, Roberta Ciarapica, Antonio F. Campese, Isabella Screpanti, Stefano Stifani, Jan Kitajewski, Lucio Miele, Rossella Rota, Franco Locatelli

**Affiliations:** ^1^ Department of Oncohematology, Ospedale Pediatrico Bambino Gesù, IRCCS, Rome, Italy; ^2^ Department of Sorbonne Universités, UPMC University Paris 06, CNRS, UMR, IBPS, Paris, France; ^3^ Liver Research Unit, Ospedale Pediatrico Bambino Gesù, IRCCS, Rome, Italy; ^4^ Department of Unit of Flow Cytometry, Ospedale Pediatrico Bambino Gesù, IRCCS, Rome, Italy; ^5^ Department of Molecular Medicine, Sapienza University, Rome, Italy; ^6^ Center for Neuronal Survival, Montreal Neurological Institute, McGill University, Montreal, QC, Canada; ^7^ Departments of Pathology and Ob/Gyn, Columbia University Medical Center, New York, NY, USA; ^8^ Department of Genetics and Stanley Scott Cancer Center, Louisiana State University Health Sciences Center and Louisiana Cancer Research Consortium, New Orleans, LA, USA; ^9^ Dipartimento di Scienze Pediatriche, Università di Pavia, Pavia PV, Italy

**Keywords:** Notch1, Notch4, endothelial cells, IL-1β, VCAM1, Pathology Section

## Abstract

The Notch1 and Notch4 signaling pathways regulate endothelial cell homeostasis. Inflammatory cytokines induce the expression of endothelial adhesion molecules, including VCAM1, partly by downregulating Notch4 signaling. We investigated the role of endothelial Notch1 in this IL-1β-mediated process. Brief treatment with IL-1β upregulated endothelial VCAM1 and Notch ligand Jagged1. IL-1β decreased Notch1 mRNA levels, but levels of the active Notch1ICD protein remained constant. IL-1β-mediated VCAM1 induction was downregulated in endothelial cells subjected to pretreatment with a pharmacological inhibitor of the γ-secretase, which activates Notch receptors, producing NotchICD. It was also downregulated in cells in which Notch1 and/or Jagged1 were silenced.

Conversely, the forced expression of Notch1ICD in naïve endothelial cells upregulated VCAM1 *per se* and amplified IL-1β-mediated VCAM1 induction. Jagged1 levels increased and Notch4 signaling was downregulated in parallel. Finally, Notch1ICD and Jagged1 expression was upregulated in the endothelium of the liver in a model of chronic liver inflammation.

In conclusion, we describe here a cell-autonomous, pro-inflammatory endothelial Notch1-Jagged1 circuit (i) triggering the expression of VCAM1 even in the absence of inflammatory cytokines and (ii) enhancing the effects of IL-1β. Thus, IL-1β regulates Notch1 and Notch4 activity in opposite directions, consistent with a selective targeting of Notch1 in inflamed endothelium.

## INTRODUCTION

Notch signaling is an evolutionarily conserved pathway that modulates cell fate decisions through local cell-cell interactions [1]. In mammals, four type I transmembrane receptors, Notch1-4, and 5 Delta-Serrate ligands, Delta-like1 (Dll1), Dll3- 4 and Jagged1-2, have been identified [1, 2]. Notch activation mostly occurs when cell-surface ligands bind to Notch receptors on adjacent cells, triggering two consecutive proteolytic cleavages of the receptor, the second of which is mediated by the γ-secretase complex. The active protein, the cleaved Notch intracellular domain (NotchICD), relocates to the nucleus, where it interacts with the DNA-binding protein RBP-Jk, activating a transcriptional complex known as CSL (for CBF1/Su(H)/Lag1) and containing mastermind-like (MAML) 1-3. The CSL-NotchICD complex then recruits co-activators, to induce the expression of canonical Notch target genes [3]. These genes include members of the Hes (Hairy and enhancer of split) and HRT/Hey (Hairy-related transcription) gene families encoding bHLH transcriptional regulators acting as repressors [3]. Non-canonical Notch signaling, involving the activation of NF-κB signaling and other pathways, has been described in several systems [4, 5]. All Notch paralogs can signal through similar pathways, but paralog-specific downstream effects have also been described [6–12].

The expression and activation of Notch signaling components are strictly tissue- and context-specific [13], complicating the situation even further. In the vascular bed, in addition to Dll1, Dll4, Jagged1 and Jagged2, endothelial cells also express both Notch1 and Notch4. The presence of Notch1has been reported in several other tissues, but Notch4 is present almost exclusively in the endothelium [14–21].

During the postnatal period, Notch signaling regulates a plethora of functions, including a number of inflammatory processes in different types of tissue [22–29].

Inflammation triggers the upregulation of vascular cell adhesion molecule 1 (VCAM1) and E-selectin expression in endothelial cells. These two adhesion molecules promote the accumulation of leukocytes and their adhesion to blood vessel walls. This phenomenon is mediated by inflammatory cytokines, such as TNFα and IL-1β, through the activation of NF-kB and downregulation of Notch4 signaling [26]. Accordingly, decreases in the expression of Notch4 and/or its target gene, Hes1, are sufficient to trigger endothelial VCAM1 expression, even in the absence of inflammatory cues [26]. TNFα and IL-1β have recently been shown to increase expression of the Notch2 receptor in endothelial cells, inducing apoptosis [28, 30]. These findings suggest that Notch receptors may have paralog-specific effects in inflamed endothelium.

The role of Notch1 in the endothelial compartment during inflammation remains unclear. We show here, in different types of endothelial cells, that Notch1: i) induces the expression of adhesion molecules, such as VCAM1, even in the absence of inflammatory cytokines, and ii) potentiates IL-1β-dependent VCAM1 upregulation, presumably through Jagged1 overexpression and with a mechanism involving the NF-kB pathway. These findings provide evidence for a novel cell-autonomous pro-inflammatory Jagged1-Notch1 pathway in endothelial cells, different from and acting in the opposite direction to the anti-inflammatory Notch4 signaling pathway occurring in the same cellular context.

## RESULTS

### IL-1β modulates Notch pathway components in endothelial cells

**Figure 1:**
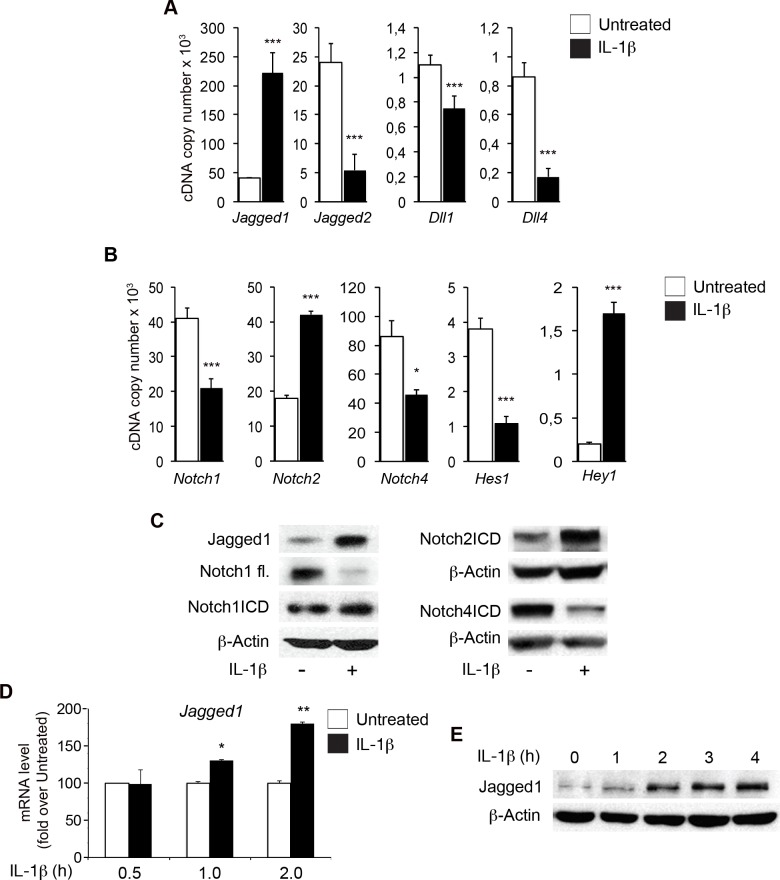
IL-1β modulates the expression of components of the Notch pathway in human aortic endothelial cells (HAECs) Confluent primary HAECs were treated with IL-1β (10 ng/ml) for 6 h or left untreated. **A.** mRNA levels (Sybr Green method, as reported in the Methods section) for Notch ligands were normalized with respect to β-actin and are expressed as the cDNA copy number (x10^3^) (see Methods section). **B.** mRNA levels (Sybr Green method) for the Notch receptors Notch1, Notch2, Notch4 and for the Notch target genes *Hes1* and *Hey1* were normalized with respect to β-actin and are expressed as the cDNA copy number (x10^3^) (see Methods section). **A.** and **B.**: Mean±SD. **C.** Representative western blot showing levels of Jagged1, full-length Notch1 (FL) and the Notch1 intracellular domain (Notch1ICD) (left), Notch2ICD and Notch4ICD (right) in HAECs treated for 6 h with IL-1β or left untreated. β-Actin is the loading control. **D.** mRNA levels for Jagged1 in HAECs treated with IL-1β or left untreated for the reported times, quantified by the 2^(−ΔΔCt)^ method (see Methods section) after normalization with respect to β-actin, expressed as a fold-change with respect to untreated sample at 0.5 h (100 arbitrary units). Mean±SEM. **E.** Representative western blot showing levels of Jagged1 in HAECs treated with IL-1β for the reported times. β-actin is the loading control. All experiments were performed in duplicate and repeated independently at least 3 times. *P < 0.05, **P < 0.01, ***P < 0.001, *t*-test.

We analyzed the role of endothelial Notch1 in early inflammation, by treating human aortic endothelial cells (HAECs) with 10 ng/ml IL-1β for 6 h [31]. IL-1β downregulated the levels of transcripts for the Notch ligands Jagged2, Dll1 and Dll4 and markedly upregulated those of the Jagged1 transcript (Figure 1A). As previously reported [26, 28], the levels of Notch1 and Notch4 transcripts decreased whereas those of the Notch2 transcript increased (Figure 1B). Levels of Jagged1 protein and of the cleaved active form of Notch2 (Notch2ICD) increased markedly, whereas those of Notch4ICD decreased (Figure 1C). Interestingly, levels of the full-length Notch1 protein were decreased by IL-1β treatment, whereas those of Notch1ICD remained similar to the levels in untreated cells (Figure 1C). These findings suggest that IL-1β activity underlies the activation of Notch1.

As previously reported for TNFα, mRNA levels for the canonical Notch target gene Hes1 were strongly decreased, whereas those for Hey1 were markedly increased by IL-1β treatment (Figure 1B).

Substantial upregulation of Jagged1 levels by IL-1β was observed within one hour of treatment (Figure 1D, 1E), consistent with previous reports [22, 27]. Similar effects of IL-1β were detected in human endothelial cells from different sources, including umbilical vein (HUVEC) and microvascular endothelial cells (HMVEC), and in cord blood endothelial progenitors (EPCs) (Supplementary Figure 1), suggesting that the effects of cytokines on the expression of Notch components are conserved between different types of endothelial cells.

### Pharmacological inhibition of Notch signaling impairs IL-1β-induced VCAM1 expression in endothelial cells

**Figure 2:**
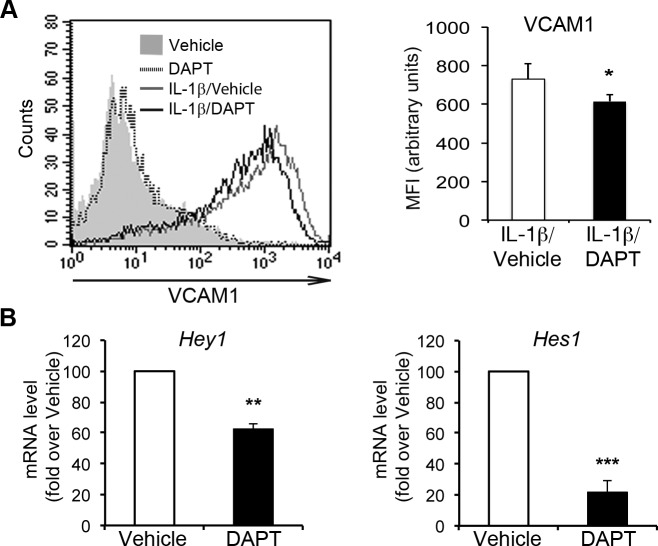
Pharmacological inhibition of Notch signaling impairs IL-1β-induced VCAM1 upregulation in human aortic endothelial cells (HAECs) Confluent HAECs were subjected to pretreatment for 16 h with either the γ-secretase inhibitor DAPT (5 μM) or vehicle (DMSO), and were then treated for six hours with DAPT and IL-1β (10 ng/ml) or with DAPT alone and subjected to flow cytometry analysis. (**A.,** left) A representative histogram shows overlays of VCAM1 expression analyzed by flow cytometry: Vehicle (gray solid curve), DAPT (dotted line), IL-1 β + Vehicle (gray line) and IL-1β + DAPT (black line). (**A.**, right) The histogram depicts the quantification of VCAM1 expression in IL-1β-treated cells, expressed as a mean fluorescence intensity (MFI), in arbitrary values. Mean±SD. **B.** mRNA levels of *Hey1* (left) and *Hes1* (right) in cells 16 h after treatment with either DAPT (5 μM) or vehicle (DMSO) were quantified by the 2^(−ΔΔCt)^ method (see Methods section) after normalization with respect to β-actin, and are expressed as a fold-change relative to vehicle-treated cells (100 arbitrary unit). Mean±SEM. All the experiments were performed in duplicate and repeated independently at least 3 times. *P < 0.05, **P < 0.01, ***P < 0.001, *t*-test.

We then investigated the role of Notch signaling in the IL-1β-dependent endothelial “activated” phenotype characterized by upregulation of the adhesion molecules VCAM1, ICAM1 and E-selectin [32]. We blocked the activation of Notch receptors in HAECs with the γ-secretase inhibitor DAPT (N-[N-(3,5-difluorophenacetyl)-l-alanyl]-S-phenylglycine t-butyl ester), which prevents the activation/cleavage of Notch receptors in a non-selective manner. HAECs treated with 5 μM DAPT for 16 h and then cotreated with DAPT and IL-1β for a further 6 h displayed a lower level of VCAM1 upregulation than cells treated with IL-1β alone (Figure 2A). With such pretreatment, DAPT downregulated the Notch targets Hey1 and Hes1, confirming the inhibition of canonical Notch activity (Figure 2B). In the absence of IL-1β, DAPT had no effect on VCAM1 expression. These results suggest that the activation of one or more Notch receptors may contribute to the endothelial induction of VCAM1 in an inflammatory context.

### The silencing of Notch1 and/or Jagged1 counteracts the IL-1β-dependent upregulation of adhesion molecule expression in endothelial cells

**Figure 3:**
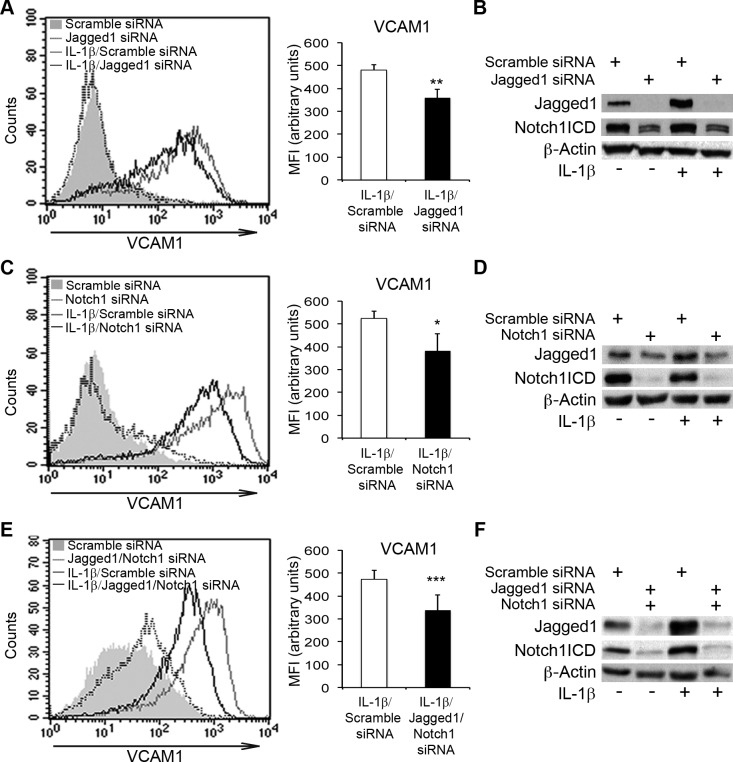
The silencing of Notch1 and Jagged1 impairs IL-1β-induced VCAM1 upregulation in human aortic endothelial cells (HAECs) (**A.**, left) HAECs were transiently transfected with either small interfering oligo RNAs (siRNAs) targeting Jagged1 (375 nM) or non-targeting control siRNAs (Scramble siRNA) and, 48 h later, they were treated with IL-1β (10 ng/ml) for 6 h or left untreated. The cells were then harvested and analyzed by flow cytometry. Representative histogram showing overlays of VCAM1 expression analyzed by flow cytometry: Scramble siRNA (gray solid curve), Jagged1 siRNA (dotted line), IL-1β + Scramble siRNA (gray line) and IL-1β + Jagged1 siRNA (black line). (**A.**, right) The histogram depicts the quantification of VCAM1 expression in IL-1β-treated cells analyzed by flow cytometry and expressed as a mean fluorescence intensity (MFI) in arbitrary values. Mean±SD. **B.** Representative western blot showing levels of Jagged1 and Notch1 intracellular domain (Notch1ICD) in HAECs treated as in **A.** β-actin is the loading control. (**C.**, left) HAECs were transiently transfected with either a siRNA targeting Notch1 (375 nM) or non-targeting control siRNAs (Scramble siRNA) and, 48 h later, they were treated with IL-1β (10 ng/ml) or left untreated for 6 h, harvested and analyzed by flow cytometry. A representative histogram showing overlays of VCAM1 expression analyzed by flow cytometry: Scramble siRNA (gray solid curve), Notch1 siRNA (dotted line), IL-1β + Scramble siRNA (gray line) and IL-1β + Notch1 siRNA (black line). (**C.**, right) The histogram depicts the quantification of VCAM1 expression in IL-1β-treated cells analyzed by flow cytometry and expressed as a mean fluorescence intensity (MFI) in arbitrary values. Mean±SD. **D.** Representative western blot showing levels of Jagged1 and Notch1 intracellular domain (Notch1ICD) in HAECs treated as in **C.** β-actin is the loading control. (**E.**, left) HAECs were transiently cotransfected with siRNAs targeting either Jagged1 (125 nM) or Notch1 (250 nM), or with non-targeting control siRNAs (Scramble siRNA) and, 48 h later, they were treated with IL-1β (10 ng/ml) for 6 h or left untreated. They were then harvested and analyzed by flow cytometry. A representative histogram showing overlays of VCAM1 expression analyzed by flow cytometry: Scramble siRNA (gray solid curve), Jagged1 siRNA + Notch1 siRNA (dotted line), IL-1β + Scramble siRNA (gray line) and IL-1β + Jagged1 siRNA + Notch1 siRNA (black line). (**E.**, right) The histogram depicts the quantification of VCAM1 expression, analyzed by flow cytometry, in IL-1β-treated cells and expressed as a mean fluorescence intensity (MFI), in arbitrary values. Mean±SD. **F.** Representative western blot showing levels of Jagged1 and Notch1 intracellular domain (Notch1ICD) in HAECs treated as in **E.** β-actin expression was used as the loading control. All experiments were performed in triplicate and repeated independently at least 3 times. *P < 0.05, **P < 0.01, ***P < 0.001, *t*-test

DAPT and other γ-secretase inhibitors are non-selective inhibitors of Notch receptors. We therefore transfected HAECs with siRNAs targeting either Jagged1 or Notch1, to determine the specific roles of these molecules in the IL-1β-induced upregulation of VCAM1. We used a non-targeting siRNA (Scramble siRNA) as a control. The depletion of either Jagged1 or Notch1 resulted in lower levels of VCAM1 upregulation upon IL-1β treatment than were observed with the Scramble siRNA (Figure 3A, 3C). Moreover, (i) Jagged1 siRNA decreased Notch1ICD levels (Figure 3B); (ii) Notch1 knockdown also prevented the IL-1β-dependent induction of Jagged1 (Figure 3D). Thus, in this context, Jagged1 activated Notch1, which, in turn, enhanced IL-1β-mediated Jagged1 overexpression.

Following the silencing of both Jagged1 and Notch1 in HAECs treated with IL-1β: (i) VCAM1 upregulation was inhibited (Figure 3E, 3F and Supplementary Figure 2A, 2B): (ii) the decrease in Notch4ICD and Hes1 levels was prevented (Supplementary Figure 2B) and (iii) the upregulation of both ICAM1 and E-selectin reduced (Supplementary Figure 2C, 2D). Moreover, Jagged1/Notch1 codepletion decreased Hey1 mRNA levels, suggesting that Notch1 activation triggered Hey1 expression either directly or indirectly in this context (Supplementary Figure 2E). These results support the hypothesis that Jagged1 and Notch1 participate in a mechanism sustaining the IL-1β-mediated induction of inflammatory adhesion molecules in endothelial cells. They also suggest that the persistence of Notch1ICD in IL-1β-treated endothelial cells may be required for the induction of Jagged1.

### Notch1ICD overexpression induces VCAM1 upregulation in endothelial cells

**Figure 4:**
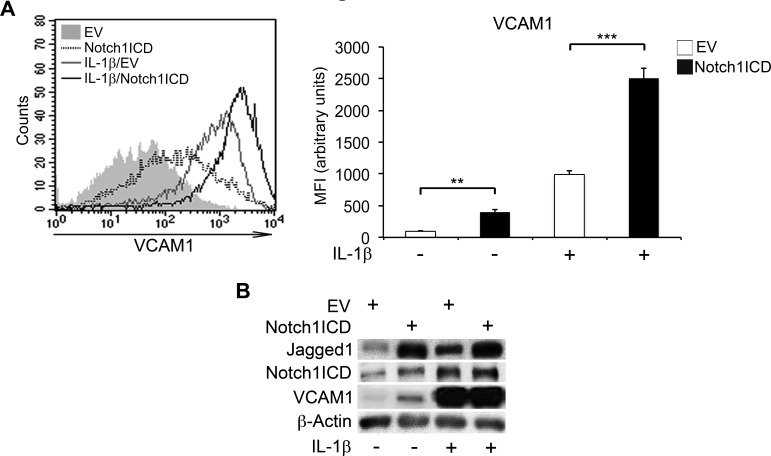
Forced expression of Notch1ICD increases VCAM1 expression in human aortic endothelial cells (HAECs) HAECs were co-nucleofected with a plasmid encoding the Notch1 intracellular domain (Notch1ICD) and a plasmid encoding the green fluorescent protein (GFP) (3:1 molar proportion) and, 48 h later, they were treated with IL-1β (10 ng/ml) for 6 h or left untreated. They were then harvested and analyzed by flow cytometry. An empty vector (EV) was used as a control. (**A.**, left) Representative histogram showing overlays of VCAM1 expression analyzed by flow cytometry: Empty Vector (EV) (gray solid curve), Notch1ICD (dotted line), Empty Vector + IL-1β (gray line) and Notch1ICD + IL-1β (black line). (**A.**, right) The histogram depicts the quantification of VCAM1 expression, analyzed by flow cytometry and expressed as a mean fluorescence intensity (MFI) in arbitrary values. Mean±SD. **B.** Representative western blot showing levels of Jagged1, Notch1 intracellular domain (Notch1ICD) and VCAM1 in HAECs treated as in **A.** β-actin is the loading control. **P < 0.01, ***P < 0.001, ANOVA (Bonferroni correction)

We used a gain-of-function approach to determine whether Notch1 activation was sufficient to trigger VCAM1 expression in HAECs. We cotransfected these cells with a plasmid encoding the human Notch1ICD and a plasmid encoding green fluorescent protein (GFP). Cells cotransfected with an empty vector (EV) and a GFP vector were used as controls (see Supplementary Figure 3A for cotransfection efficiencies). Strikingly, in the absence of IL-1β, Notch1ICD overexpression alone led to higher VCAM1 levels in HAECs than in EV-transfected cells (Figure 4A, 4B and Supplementary Figure 3B). Notch1ICD further enhanced the stimulation of VCAM expression by IL-1β treatment, as shown by comparisons with EV-transfected cells (Figure 4A, 4B). Consistent with the results of knockdown experiments, Notch1ICD expression also increased Jagged1 levels in both the presence and absence of IL-1β (Figure 4B and Supplementary Figure 3B). Notch1ICD overexpression in HAECs also had an effect similar to that of IL-1β reported in previous studies by our group and others [26, 30]: it resulted in higher levels of Hey1 and Notch2 transcripts and lower levels of Notch4 and Hes1 transcripts than observed for EV-transfected cells (Supplementary Figure 3C).

DAPT pretreatment did not abolish Notch1ICD-dependent VCAM1 overexpression in HUVECs infected with a retroviral vector encoding the mouse Notch1ICD (Notch1ICD*) previously validated by our group [33] (Supplementary Figure 4), suggesting that the observed effects were specific to Notch1.

Thus, in the absence of other activated Notch paralogs, Notch1ICD was able to mimic the effects of IL-1β on endothelial VCAM1 expression.

### The effects of Notch1ICD overexpression on VCAM1 in endothelial cells are partly counteracted by the pharmacological inhibition of NF-kB

**Figure 5:**
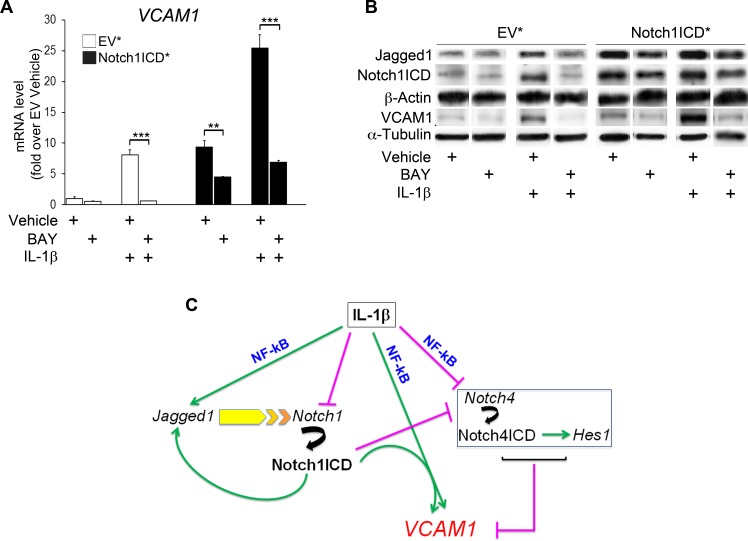
Notch1ICD-mediated VCAM1 induction is partly counteracted by NF-kB inhibition in human umbilical vein endothelial cells (HUVECs) HUVECs were transduced with either a retroviral vector co-expressing a flag-tagged murine Notch1ICD (Notch1ICD*) and the enhanced green fluorescent protein (eGFP) or an eGFP vector (EV*) as a control. After 48 h from infection, cells were subjected to pretreatment with the NF-kB inhibitor BAY 11-7082 (20 μM) for 1 h or with vehicle (DMSO) and they were then treated with IL-1β for 1 h or left untreated. **A.** Levels of VCAM1 mRNA were quantified by the 2^(−ΔΔCt)^ method (see Materials and Methods section) after normalization with respect to β-actin, and expressed as a fold-change relative to EV*-vehicle-treated cells (1 arbitrary unit). Mean±SD. **B.** Representative western blot showing levels of intracellular domain (Notch1ICD), Jagged1 and VCAM1 in HUVECs treated as in **A.** α-tubulin is the loading control. The experiments were performed independently and repeated at least twice. **P < 0.01, ***P < 0.001, ANOVA (Bonferroni correction). **C.** Proposed model of Notch1-dependent induction of endothelial VCAM1. In endothelial cells, IL-1β reduces the transcription of the Notch4 gene in an NF-kB-dependent manner, leading to downregulation of both the active Notch4ICD form and expression of the Notch4 target gene *Hes1*. Both these both phenomena are responsible, at least in part, for the upregulation of VCAM1 in the endothelium. Concomitantly, IL-1β induces VCAM1 expression in an NF-kB-dependent manner. In parallel, IL-1β decreases Notch1 transcript levels and upregulates expression of the Notch ligand Jagged1 via a mechanism involving NF-kB. Jagged1 binds to Notch1, leading to the cleavage of the receptor and the sustained formation of the activated form Notch1ICD, in turn favoring Jagged1 expression. At the same time, Notch1ICD amplifies the NF-kB-dependent VCAM1 expression induced by IL-1β. Possibly due to Notch1-inducing VCAM1 upregulation in the absence of IL-1β, the activation of Notch1 also decreases Notch4 and Hes1 expression.

NF-kB activation has been implicated in the TNFα-mediated induction of endothelial VCAM1 [26, 27, 34]. We investigated the contribution of NF-kB signaling to the induction of VCAM1 expression by Notch1, by subjecting Notch1ICD*-overexpressing HUVECs to pretreatment for 1 h with the NF-kB inhibitor BAY 117082 (BAY; 20 μM) before IL-1β treatment. BAY pretreatment abolishes NF-kB phosphorylation and signaling by inhibiting IkB kinase (IKK) activity [35], thereby blocking inflammatory VCAM1 expression in endothelial cells [34]. As expected, BAY pretreatment completely prevented the increase in VCAM1 levels in empty vector-transfected (EV*) cells treated with IL-1β (Figure 5A, 5B). It also attenuated the derepression of VCAM1 by Notch1ICD* in HUVECs in the absence of IL-1β.

Moreover, even though Notch1ICD strongly amplified the increase in VCAM1 expression in response to the cytokine, BAY pretreatment markedly inhibited this phenomenon (Figure 5A, 5B). Finally, BAY also decreased the induction of Jagged1 under all experimental conditions tested (Figure 5B).

These results confirm that endothelial Notch1 activation enhances VCAM1 expression by a mechanism involving NF-kB activation.

### Jagged1 and Notch1 are upregulated in rat liver vessels with low-grade chronic inflammation

**Figure 6:**
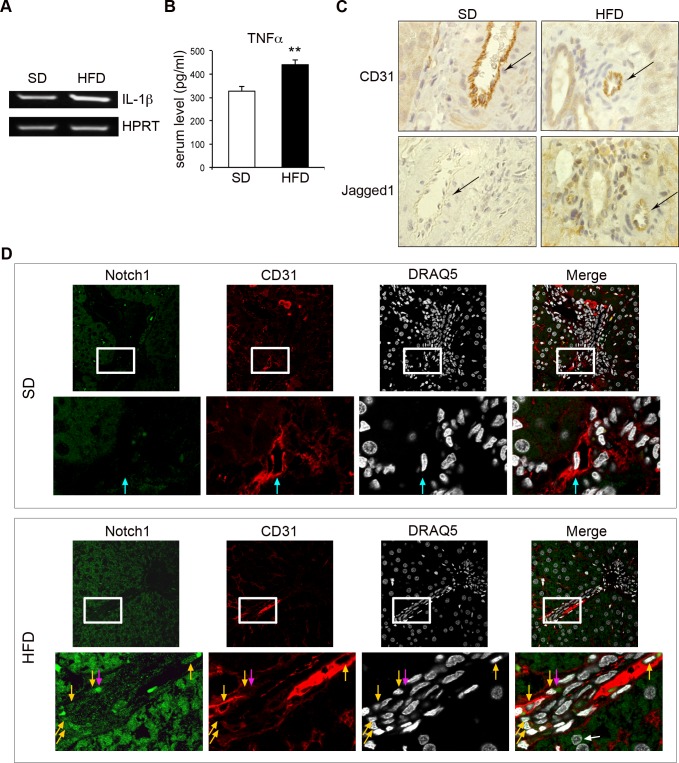
Jagged1 and Notch1 are upregulated in the liver vessels of rats with low-grade chronic inflammation Rats were fed *ad libitum* with either a high-fat diet (HFD) or standard chow (SD) (control rats) and were analyzed after 3 months. **A.** Representative semi-quantitative RT-PCR for *IL-1β* mRNA, on total RNA from the livers of SD- or HFD-fed rats. Following the preliminary setting up of PCR conditions, each sample was analyzed during the linear phase of amplification and PCR products were visualized under UV illumination with gel red (Biotium Inc., Hayward, CA), after electrophoresis in a 1.5% agarose gel. Primer sequences are listed in Table 1. HPRT was used as the housekeeping gene. **B.** Quantification by an enzyme-linked immunosorbent assay (ELISA) for TNFα in the serum of SD- and HFD-fed rats. **C.** Immunohistochemical staining showing CD31 (top) and Jagged1 (bottom) expression on endothelial cells from serial sections of liver samples from SD- and HFD-fed rats. Positive cells are indicated by brown staining. The arrows indicate the endothelial cells lining the blood vessels. 600x magnification. **D.** Double-labeling immunofluorescence analysis of Notch1ICD (green), with an anti-Val1744 antibody that detects only the cleaved form of the receptor, and CD31 (red) on serial sections of liver from SD- and HFD-fed rats. Nuclei are counterstained with DRAQ5. Blue arrows indicate Notch1ICD-negative nuclei (SD rats), yellow arrows indicate Notch1ICD-positive nuclei and pink arrows indicate Notch1ICD-positive cytoplasmic staining (HFD rats). A white arrow indicates a Notch1ICD-positive liver parenchymal nucleus. Top panels, 600 x magnification; lower panels are a higher magnification (2400 x magnification) of the squared area in the top panels. A representative set of images is shown. **P < 0.01 *t*-test.

We investigated the possible deregulation of Jagged1 and Notch1 in inflamed endothelial cells *in vivo*, in an animal model of low-grade chronic inflammation. We used the high-fat diet (HFD)-rat model, in which the histological features of the liver resemble those of human non-alcoholic fatty liver disease (NAFLD), as previously demonstrated by our group [36]. NAFLD is a common, chronic inflammatory liver disease associated with obesity and characterized by a pattern of steatosis associated with low-grade CD163-positive inflammation. HFD rats have recently been reported to have high serum TNF-α levels [37] and to display positive hepatic staining for IL-1β [38], which was absent in normal control animals. Consistent with these observations, IL-1β mRNA levels in the livers of HFD rats were 70% higher than those in controls (Figure 6A). Serum TNF-α levels were also considerably higher in HFD rats [37] (Figure 6B).

Immunohistochemical analysis showed that Jagged1 was expressed by the CD31-positive cells lining the liver sinusoids and a few hepatocytes in HFD rats, but not in control rats (Figure 6C). Staining for nuclear Notch1ICD with an anti-Val1744 antibody recognizing only the cleaved form of the receptor [11] showed this protein to be present in hepatocytes, as previously reported [39], and in the liver endothelial cells of HFD rats, whereas it was almost undetectable in controls (Figure 6D).

These results are consistent with the notion that low-grade chronic inflammation of the liver is associated with increases in Jagged1 expression and Notch1 activation in endothelial cells.

## DISCUSSION

Our results build on previous reports concerning the role of Notch signaling in an endothelial inflammatory context. They reveal, for the first time, that Notch1 activation in endothelial cells mimics the effects of IL-1β on adhesion molecules and on other Notch signaling components. Indeed, we found that (i) the active form Notch1ICD was sufficient to induce VCAM1 and Jagged1 expression in the absence of IL-1β, this induction being markedly amplified by the presence of this cytokine, partly through NF-kB, with this active form also enhancing the induction of expression induced by IL-1β, and (ii) Notch1 knockdown inhibited this phenomenon.

Consistent with these findings, Notch1 remained activated in the early phase of IL-1β treatment, whereas Notch4 did not, despite the decrease in Notch1 mRNA and full-length precursor protein levels induced by IL-1β. This appears to be due, in part, to the IL-1β-dependent increase in Jagged1 levels [27, 40–42], because Jagged1 silencing decreased Notch1 activation and was associated with a decrease in VCAM1 expression.

These results suggest that there is a cell-autonomous positive feedback loop between Notch1 and Jagged1 during the early stages of inflammation, sustaining Notch1 signaling in endothelial cells and contributing to the effects of IL-1β on VCAM1 expression. In later phases of the inflammatory response, Notch1 may be required for the sustained induction of Jagged1 [27, 43, 44].

Despite the decrease in Notch4 signaling caused by IL-1β in inflamed endothelium, the decrease in VCAM1 levels following pretreatment with the γ-secretase inhibitor DAPT, a non-selective inhibitor of Notch receptor activation, confirmed that Notch activation facilitated the endothelial inflammatory phenotype.

### Crosstalk between Notch1 signaling and NF-kB in endothelial cells

Our observation that the NF-kB inhibitor BAY markedly decreased Notch1ICD and Jagged1 levels in cells treated with IL-1β suggests that, in this context, the Notch1-Jagged1 positive feedback loop involves Notch1 signaling through NF-kB. Our results are consistent with previous reports that activated NF-kB induces Jagged1, which then can trigger Notch signaling transcriptional activity, in endothelial cells [27]. By contrast, the pretreatment of Notch1ICD cells with BAY only partially inhibited the enhancement of both VCAM1 and Jagged1 expression in the presence or absence of IL-1β. This indicates that high levels of Notch1ICD in endothelial cells may be involved in NF-kB-independent signaling.

Several mechanisms may underlie the observed phenomenon involving NF-kB and Notch1 [5, 43, 45], including the occupancy of nearby chromatin sites by NF-kB and Notch1ICD/RBP-jK [46, 47] and the presence of an RBP-jK binding site nested within an NF-kB binding site [48].

Consistent with these findings, the NF-kB and Notch1 signaling pathways cooperate in the stimulation of Jagged1 expression in macrophages, but can also act independently [43]. However, in light of our results and recent reports on the participation of these transcriptional regulators in super-enhancers [34, 49], further investigations of the chromatin crosstalk between Notch1 and Jagged1 in the inflamed endothelium are required.

### Notch1 regulates the expression of Notch components in endothelial cells

Like IL-1β, Notch1ICD modulates other Notch signaling components, decreasing Notch4 and Hes1 levels, whereas Notch1 knockdown increases the levels of these components. The opposite is true for Hey1, suggesting that Notch4 signals through Hes1 in endothelial cells, contrary to reports for other systems [50]. Previous data have shown that the silencing of Notch4, unlike that of Notch1, does not modulate the transcription of genes encoding other Notch paralogs under the same experimental conditions [26, 28].

It is not possible to determine whether Hey1 is responsible for repressing Notch4 expression from our data. However, TNFα-mediated Hey1 induction has been shown to result from Notch2ICD overexpression [28]. As activated Notch1 strongly induces Notch2 expression (our data and [28, 30]), the upregulation of Hey1 may be dependent on Notch2, which does not regulate the expression of adhesion molecules ([28, 30] and our unpublished observations).

An example of opposite patterns of regulation by IL-1β has been reported for two Notch paralogs in the vascular bed, with this cytokine inducing Notch1 and Hey1 and downregulating Notch3 and Hes1 in vascular smooth muscle cells (VSMCs) [23].

We also showed that the expression of Notch1ICD and Jagged1 was upregulated in the liver endothelial cells of rats with hepatic chronic inflammation (i.e., NAFLD), as previously reported for hepatocytes [39]. This result is consistent with the notion that endothelial Notch1 is activated by inflammatory cytokines, such as IL-1β and TNFα, *in vivo* and suggests that Notch1 may play a role in chronic endothelial inflammation.

## CONCLUSIONS

In summary, we describe a novel pro-inflammatory role for endothelial Notch1, opposing that of Notch4 (Figure 5C), and suggest that there may be an endothelial Notch1-Jagged1 cell-autonomous circuit supporting the endothelial inflammatory phenotype and involving NF-kB.

Notch1 has already been implicated in endothelial functions and in vascular biology in general, but our results highlight, for the first time, the involvement of this Notch paralog in the regulation of endothelial inflammatory adhesion molecules. The mechanisms underlying such paralog-specific effects and their possible pathophysiological relevance require further investigation, to improve the precision of Notch signal modulation in inflamed endothelia [50].

## MATERIALS AND METHODS

### Cell culture and reagents

Primary human arterial endothelial cells (HAECs) and primary human umbilical vein endothelial cells (HUVECs) were purchased from Lonza Group Ltd. (Basel, Switzerland), cultured in EGM-2 complete medium (Lonza Group Ltd, Basel, Switzerland), and used between passages 4 and 7. Confluent cell monolayers were treated with IL-1β (Peprotech, Rocky Hill, NJ) 10 ng/ml in EBM-2 (Lonza Group Ltd, Basel, Switzerland) supplemented with 0.5% FCS (Hyclone, Logan, UT); DAPT and BAY 11-7082 (both from Sigma Chemical Co., St Louis, MO) were dissolved in DMSO and used at concentrations of 5 μM or 10 μM (DAPT) and 20 μM (BAY 11-7082) for the indicated times.

### Transient RNA interference and transfection with plasmids

Endothelial cells were transfected with siRNAs against Jagged1 and Notch1 (Sasi_Hs01_00100442 and Sasi_Hs01_00052328, respectively; Sigma Chemical Co., St Louis, MO), in the presence of Oligofectamine (Invitrogen Corp., Carlsbad, CA) in Opti-MEM, at a final concentration of 375 nM, following the preliminary setup recommended by the manufacturer. For double-knockdown experiments, cells were cotransfected with 125 nM and 250 nM siRNA for Jagged1 and Notch1, respectively, this combination having been found to yield the best simultaneous silencing for both molecules (data not shown).

A non-targeting FITC-conjugated siRNA (Scramble) was used as a control for silencing (Sigma Chemical Co., St Louis, MO). For overexpression studies, pCMV-GFP was used in a molar proportion of 1:3 with a pcDNA3 vector encoding human Notch1ICD or the empty vector (a gift from M. Bocchetta, Loyola University of Chicago [10]). Nucleofection was performed with an Amaxa Nucleofector (Lonza Group Ltd, Basel, Switzerland), with the Amaxa basic nucleofector kit for primary endothelial cells (Lonza Group), according to the manufacturer's instructions. Assays were performed 48 h after transfection.

### Western blot analysis

Cells were lysed in RIPA buffer supplemented with protease inhibitor cocktail (Roche Life Science, Roche Diagnostics S.p.A. Monza (MB), Italy), sonicated for 30 minutes on ice, and the lysate was then centrifuged (20,000 x *g*, 20 min). The protein concentration of samples was determined by the BCA method (Pierce, Rockford, IL), according to the manufacturer's instructions. Proteins (40 μg) were run on SDS-polyacrylamide gels and blotted onto nitrocellulose membranes, which were blocked by incubation with 10% non-fat milk at room temperature for 1 h. The membranes were probed with primary antibodies (Jagged1 1:1000, Notch1 (bTAN) 1:1000, Notch2 1:1000 (DSHB, Iowa City, IA), Notch4 1:1000 (R&D Systems Europe, Ltd., Abingdon, UK), VCAM1 1:1000 and β-actin 1:20000 (Santa Cruz Biotechnology, Inc., Santa Cruz, CA), and an antibody against Notch1ICD cleaved at Val1744, recognizing both human and murine Notch1ICD (Cell Signaling, Beverly, MA)), by incubation overnight at 4°C with the appropriate antibody in TBS, 0.05% Tween 20, 5% milk. The membrane with then incubated with a horseradish peroxidase-conjugated secondary antibody (Santa Cruz Biotechnology) for 1 h at room temperature, and enhanced chemiluminescence (Amersham, GE Healthcare, UK) was performed according to kit manufacturer's instructions.

### Reverse transcriptase-quantitative polymerase chain reaction (RT-qPCR)

RNA was extracted with TRIzol reagent (Invitrogen), according to the manufacturer's instructions. The RNA was treated with DNase I and used as a template for generation of the first-strand cDNA by Super Script II Reverse Transcriptase (Invitrogen), according to the manufacturer's instructions. Quantitative PCR assays were performed with either the Sybr Green method (Figure 1 and Supplementary Figures 1) or *Taq*Man assays (Figures 2 and 5, and Supplementary Figures 3-5; Applied Biosystems, Life Technologies, Carlsbad, CA). With the Sybr Green method, samples were analyzed in the linear phase of amplification, as previously described [23]. The primer sequences used to amplify the desired cDNAs are detailed in Supplementary Table 1. The PCR templates consisted of 250 ng of cDNA or purified standard DNA. Amplification was performed with a spectrofluorometric thermal cycler (LightCycler 480, Roche Diagnostics, Meylan, France). After the initial denaturation step at 95°C for 5 min, amplification was performed with 50 cycles of denaturation (95°C for 10 s), annealing (60°C for 15 s) and extension (72°C for 10 s). For each run, a standard curve was generated from purified DNA, ranging from 10 to 10^6^ copies, and samples were quantified with the C_T_, by interpolation from the standard curve to yield a copy number for the cDNA corresponding to each gene. Gene expression values were normalized by dividing the copy number of the target gene by that of the β-actin gene, used as a housekeeping gene [23].

Quantitative PCR was performed with the following *Taq*Man probes: VCAM1 (Hs01003372_m1), Hey1 (Hs01114113_m1), Hes1 (Hs00172878_m1), Jagged1 (Hs01070036_m1), Notch1 (Hs01062014_m1), Notch2 (Hs01050719_m1), Notch4 (Hs00965882_m1) and β-actin (Hs99999903_m1), used as a housekeeping gene (Applied Biosystems, Life Technologies, Carlsbad, CA), with an Applied Biosystems 7900 system. The fold-change in gene expression was calculated by the 2^(−ΔΔCt)^ method [51]. At least three independent amplifications were performed for each gene, with samples analyzed at least in duplicate.

### Fluorescence-activated cell sorting (FACS) analysis

For antigen detection by FACS, cell monolayers were detached with Versene (Invitrogen Corp., Carlsbad, CA), washed and incubated with the primary antibodies against VCAM1 (BD Pharmingen, San Jose, CA), ICAM1 (Invitrogen Corp., Carlsbad, CA) and E-selectin (Upstate Biotechnology, Lake Placid, NY), or isotype-matched IgG antibodies as negative controls, diluted (1:20) in PBS 5% FCS and for 30 minutes on ice. Cy5-conjugated anti-mouse secondary IgG (Jackson Immunoresearch Laboratories, PA) diluted (1:100) in PBS were incubated with the cells for 20 minutes on ice. Acquisition was performed on a FACS LSR1 Cytometer (BD Biosciences, San Jose, CA) with MacIntosh CellQuest Pro software.

### Retroviral infection

The CMMP-IRES-EGFP retroviral vector expressing a flag-tagged murine Notch1ICD and the control empty CMMP-IRES-EGFP vector have been described elsewhere [33]. Phoenix Ampho cells obtained from ATCC were cultured in DMEM supplemented with 10% FBS and transiently transfected by the calcium phosphate method. Supernatants containing viral particles were collected after 48 h and used to infect HUVECs for 48 h (two rounds of 12 h infection) in the presence of 5 μg/ml polybrene (two rounds of infection). The HUVECs were then subjected to pretreatment with vehicle (DMSO) or DAPT (10 μM) for 16 h and harvested for examination. In parallel, infected HUVECs were subjected to pretreatment with either BAY 11-7082 (20 μM) or vehicle (DMSO) for 1 h, and then treated with 10 ng/ml IL-1β or vehicle (DMSO) for 1 h and then harvested for examination.

### Animal model of non-alcoholic fatty liver disease (NAFLD)

Rats were provided with free access to standard chow for 5 days and were then randomized (6 rats per group) to a standard diet group (SD) and a high-fat diet group (HFD). The standard chow was from Harlan Laboratories (Harlan Laboratories, Inc., Correzzana (MB), Italy) and the high-fat chow was purchased from Laboratorio Dottori Piccioni (Gessate (MI), Italy) and contained 58% energy from fat, 18% from protein, and 24% from carbohydrates (5.6 kcal/g), whereas the normal chow contained 5% energy derived from fat, 18% from proteins, and 77% from carbohydrates (3.3 kcal/g). All rats were killed after three months of diet. At the indicated time points, livers were excised, weighed and processed for immunohistochemical analysis or RNA extraction. Blood samples were collected for the determination of TNFα levels.

### Immunohistochemistry and immunofluorescence

Liver tissues from NAFLD and control animals were fixed by incubation in 10% formalin at room temperature for 24 h and embedded in paraffin. The specimens were cut into 2 μm section, which were deparaffinized, rehydrated and blocked. After citrate-based antigen retrieval (pH 6 and pH 9 for CD31 and Jagged1, respectively), sections were incubated with primary antibodies: 1:50 mouse monoclonal anti-CD31 (Novocastra, Newcastle Upon Tyne, UK) and 1:50 rabbit monoclonal anti-Jagged1 (Cell Signaling, Beverly, MA). The secondary antibody was provided by the Labelled Streptavidin Biotin (LSAB) kit (Dako Denmark A/S, Glostrup, Denmark). The diaminobenzidine (DAB) substrate chromogen kit (Dako Denmark A/S, Glostrup, Denmark) was used for detection. Nuclei were counterstained with Gill's hematoxylin. Positive reactions, indicated by brown staining, were observed with an Eclipse E600 microscope (Nikon Instruments, Firenze, Italy) and images were acquired with a Nikon Digital Camera DXM1200F and Lucia version 4.81 software.

Immunofluorescence was analyzed on 2 μm-thick sections obtained from formalin-fixed tissue embedded in paraffin. Antigen retrieval was performed with EDTA (pH 8) (Dako Denmark A/S, Glostrup, Denmark). Sections were then incubated with primary antibodies: 1:100 mouse monoclonal anti-CD31 (Novocastra, Newcastle Upon Tyne, UK) and 1:300 rabbit polyclonal Cleaved Notch-Val1744 (Cell Signaling, Beverly, MA). The secondary antibodies used for staining were: 1:500 Alexa Fluor 555-conjugated goat anti-mouse and 1:500 Alexa Fluor 488-conjugated goat anti-rabbit antibodies purchased from Applied Biosystems (Life Technologies, Carlsbad, CA). Nuclei were counterstained with DRAQ5 (Biostatus, Shepshed, UK) for 5 min and, after extensive washing, sections were mounted in PBS/glycerol (1:1) and covered with a coverslip. Images were acquired with an Olympus Fluoview FV1000 confocal microscope equipped with a 60x (numerical aperture: 1.42) oil objective. Optical single sections were acquired with a scanning mode format of 1024×1024 pixels, and a sampling speed of 20 μs/pixel, and 12 bits/pixel image. Fluorochrome signals were separated by the acquisition of an automated sequential collection of multi-channel images, to reduce spectral crosstalk between channels. The pinhole aperture was 1 Airy unit.

### Enzyme-linked immunosorbent assay

Serum TNFα levels were assessed by ELISA (Peprotech, Rocky Hill, NJ), according to the kit manufacturer's instructions.

### Statistics

Statistical analysis was performed with Student's *t*-test or ANOVA (with Bonferroni correction for multiple testing) for multiple comparisons. A *P*-value < 0.05 was considered significant.

## SUPPLEMENTARY MATERIAL FIGURES AND TABLE


