# Supplementary material for: Activation of an endothelial Notch1-Jagged1 circuit induces VCAM1 expression, an effect amplified by interleukin-1β
Source: Oncotarget. 2015 Dec 3;6(41):43216–29. doi: 10.18632/oncotarget.6456 (PMC4791227; doi:10.18632/oncotarget.6456)
Supplement: Supplementary file 1 [file oncotarget-06-43216-s001.pdf]

Activation of an endothelial Notch1-Jagged1 circuit induces VCAM1 expression, an effect amplified by interleukin-1β

Supplementary Material

Supplementary Table 1. Primer sequences utilized for Sybr Green qPCR in Figure 1 and Supplementary Figures 1 and 2

Gene Bank No Gene	Primer Sense Sequence 5'-3'	Primer Antisense Sequence 5'-3'	Amplicon size (bp)
NM_001101 β-Actin	CATGGGTCAGAAGGATTCCTAT	ATGTCGTCCCAGTTGGT	108
NM_017617 Notch1	GATGACCTGGGCAAGTC	CCCTGTTGTTCTGCATATCT	109
NM_024408 Notch2	CGCACTCAGCTCCTCTATC	GATTGCTTGCATCTCGGC	130
NM_00455 Notch4	CTGCCCCTCTGGTTTCACAG	CCCCCTAGCTCTGCCTCAG	220
NM_000214 Jagged1	GCTGACTTAGAATCCCTGTGTTA	AGGGTACTGTTGACTAGCTTT	133
NM_145159 Jagged2	ACAATGGAGTATTCTCGGATAG	CACAACCTCTGGTAACAAAC	129
NM_005618 Dll1	GTGATGAGCAGCATGGATT	CAGCCTGGATAGCGGATAC	101
NM_019074 Dll4	GCGAGAAGAAAGTGGACAGG	ATTCTCCAGGTCATGGCAAG	184
NM_005524 Hes1	CACGACACCGGATAAACCA	CTGGCTCAGACTTTCATTTATTC	104
NM_012258 Hey1	GCACGCCCTTGCTATGGA	GATGCGAAACCAGTCGAAC	128
NM_031512 IL-1β (Rat)	CTCGTGGGATGATGACGACG	GAATACCACTTGTTGGCTTA	150
NM_031144 HPRT (Rat)	CTGCGTGTGGCCCCTGAGGA	GACCAGAGATCAGGGAC	150

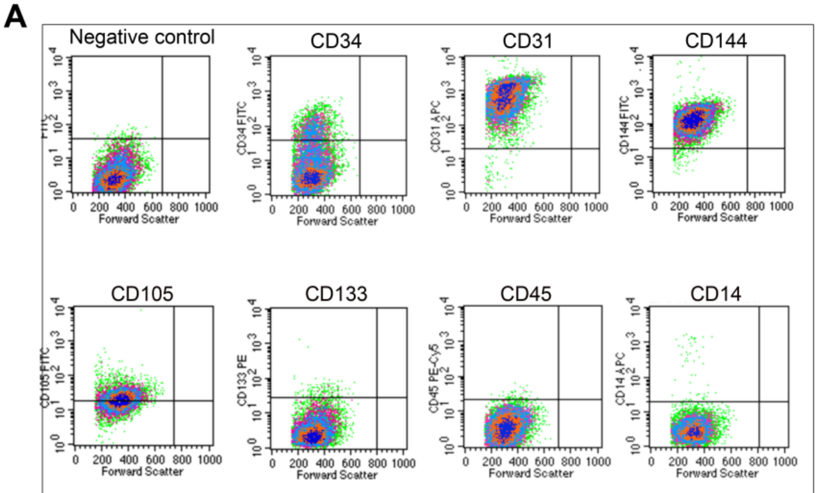

	Positive	Negative
CD34	16.23%	83.77%
CD31	99.39%	
CD144	98%	
CD133	0.39%	
CD105	57.97%	42.03%
CD45	0.09%	
CD14	0.41%	

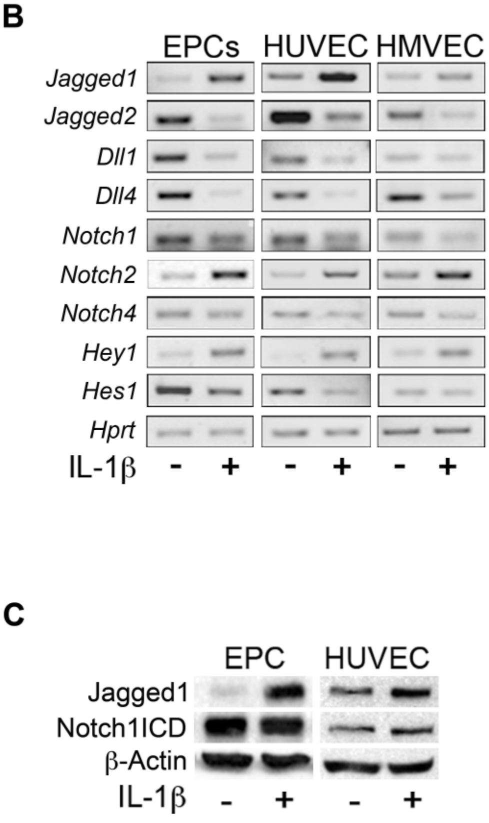

Supplementary Figure 1: IL-1β modulates mRNA levels of components of the Notch pathway in endothelial cells from different districts. Human umbilical vein endothelial cells (HUVEC) and human microvascular endothelial cells (HMVEC) were purchased from Lonza (Lonza Group Ltd., Basel, Switzerland), cultured in EGM-2 and EGM2^{MV} complete medium (Lonza Group Ltd., Basel, Switzerland) respectively, and used between passages 4 to 7. Endothelial progenitor cells (EPCs) were isolated and characterized as previously described [1], with minor modifications. Briefly, we isolated EPCs from umbilical cord blood mononuclear cells (MNCs) from healthy term newborn donors by selective method of culture. MNCs were isolated by centrifugation on Ficoll gradient and, then, cultured on collagen-coated dishes in an endothelial specific medium that selectively sustains the growth of endothelial cell lineage, i.e. EGM-2 complete medium (Lonza Group Ltd., Basel,

Switzerland). Adherent cells that gave rise to cell colonies after 10-14 days of cultures were characterized through molecular and functional assays to establish their endothelial origin. Phenotypically, EPCs-forming colonies showed the cobblestone morphology typical of endothelial cells (data not shown). (**A**) Representative flow cytometric analysis dot plots of EPCs show positivity for endothelial markers: CD34, CD31, CD144 and CD105, absence of both pan-leukocytes marker CD45, the monocyte marker CD14 and the CD133, a marker associated with hematopoietic stem cells. The table reports the percentage of Mean Fluorescence Intensity value for each marker shown in the dot plot. Also, the expression of VEGFR1, VEGFR2, Tie1 and Tie2 has been validated at mRNA level (data not shown). Functionally, both the capability to differentiate and forming capillary-like structures and the ability to up-take acetylated-low density lipoprotein (Ac-LDL) have been validated (data not shown). (**B**) Semi-quantitative RT-PCR for *Jagged1-2*, *Dll1*, *Dll4*, *Notch1*, *Notch2*, *Notch4*, *Hey1* and *Hes1* in confluent EPCs, HUVECs and HMVECs treated 6 h with IL-1β (10ng/ml) or left untreated. HPRT is used as housekeeping gene. Following a preliminary setup of PCR conditions, each sample was analyzed during linear phase of amplification and PCR products were visualized under UV illumination with gel red (Biotium Inc. Hayward, Ca) in 1.5% agarose gel. Primer sequences are listed in Table1. (**C**) Representative Western blot showing Jagged1 and Notch1ICD levels in confluent EPCs and HUVECs treated 6h with IL-1β (10ng/ml) or left untreated. β-Actin is shown as loading control. All experiments were performed in duplicates and independently repeated at least 3 times.

1. Ingram DA, Mead LE, Tanaka H, Meade V, Fenoglio A, Mortell K, Pollok K, Ferkowicz MJ, Gilley D, Yoder MC: **Identification of a novel hierarchy of endothelial progenitor cells using human peripheral and umbilical cord blood**. *Blood* 2004, **104**(9):2752-2760.

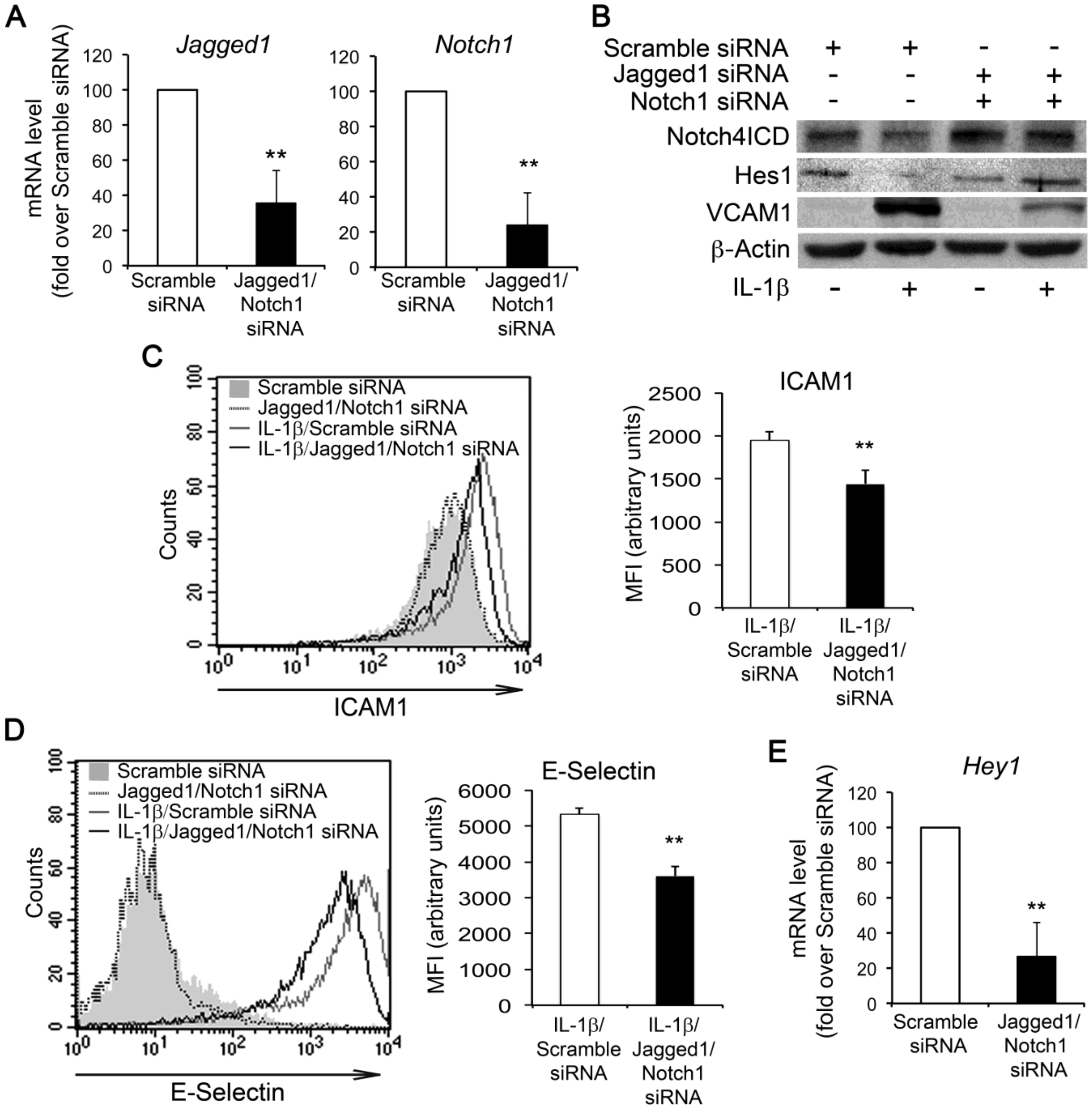

Supplementary Figure 2: Notch1 and Jagged1 silencing impaired IL-1β-induced ICAM1 and E-Selectin up-regulation in human aortic endothelial cells (HAECs). HAECs were transiently co-transfected with siRNAs targeting either Jagged1 (125nM) or Notch1 (250nM), or with a non-targeting control siRNA (Scramble siRNA) and used 48 h later for all assays. (**A**) mRNA expression levels of *Jagged1* (**left**), *Notch1* (**right**) and (**E**) *Hey1* were quantified by the $2^{(-\Delta\Delta Ct)}$ method (see Methods

section) after normalization to *β-Actin*, and expressed as fold-change over those of Scramble siRNA (100 arbitrary unit). Mean±SEM. (**B**) Forty-eight h after silencing, HAECs were treated with IL-1β (10ng/ml) for 6 h or left untreated and harvested for the following assays: Representative Western blot showing levels of Notch4 intracellular domain (Notch4ICD), Hes1 and VCAM1. β-Actin is shown as loading control. (**C-D**) HAECs treated as in (**B**) were harvested for flow cytometry analysis of ICAM1 and E-Selectin. Representative histograms show overlay of ICAM1 (**C, left**) and E-Selectin (**D, left**) expression analyzed by flow cytometry: Scramble siRNA (grey filled curve), Jagged1 siRNA + Notch1 siRNA (dotted line), IL-1β + Scramble siRNA (grey line) and IL-1β + Jagged1 siRNA + Notch1 siRNA (black line). (**C and D, right**) The histograms depict the quantification of ICAM1 (**C**) and E-Selectin (**D**) expression analyzed by flow cytometry in IL-1β -treated conditions and expressed in Mean Fluorescence Intensity (MFI) arbitrary values. Mean±SD. (**E**) In HAEC treated as in (**A**) mRNA expression levels of *Hey1* were quantified by the $2^{(-\Delta\Delta Ct)}$ method (see Methods section) after normalization to *β-Actin*, and expressed as fold-change over those of Scramble siRNA (100 arbitrary unit). Mean±SEM. All the experiments were performed in duplicates and independently repeated at least 3 times. ***P*<0.01, *t*-test.

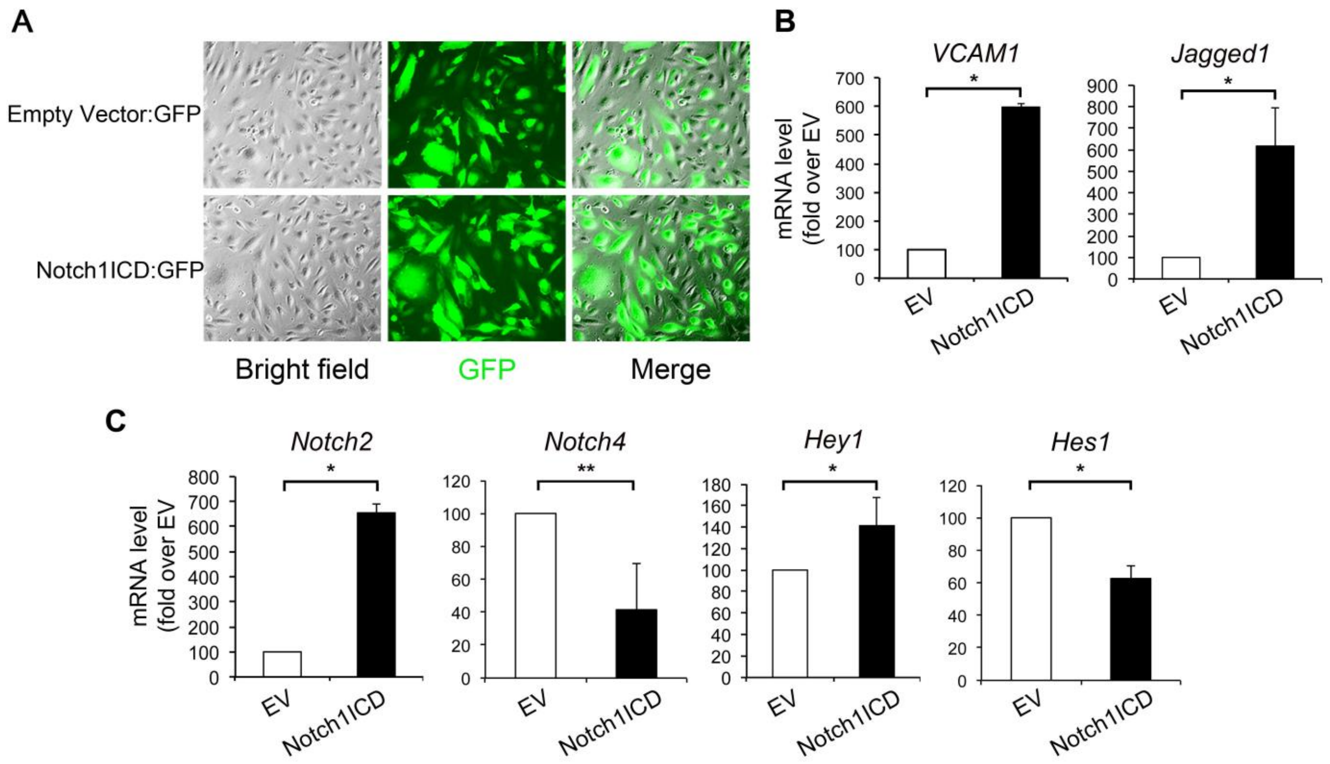

Supplementary Figure 3: Notch1ICD forced expression modulates VCAM1 and the Notch signaling components. HAECs were co-transfected by nucleofection (Amaxa) with Notch1ICD-expressing vector or an empty vector (EV) and a vector expressing GFP in molar proportions of 3:1 (see Methods section). (**A**). After 48 h of transfection cells were analyzed for the expression of GFP under a fluorescence microscope. mRNA expression levels of (**B**) *VCAM1*, *Jagged1*, and (**C**) *Notch2*, *Notch4*, *Hey1* and *Hes1* were quantified by the $2^{(-\Delta\Delta Ct)}$ method (see Methods section) after normalization to *β-Actin*, and expressed as fold-change over those of EV (100 arbitrary unit). Mean±SEM. All the experiments were performed in duplicates and independently repeated at least 3 times. *P<0.05, **P<0.01, *t*-test

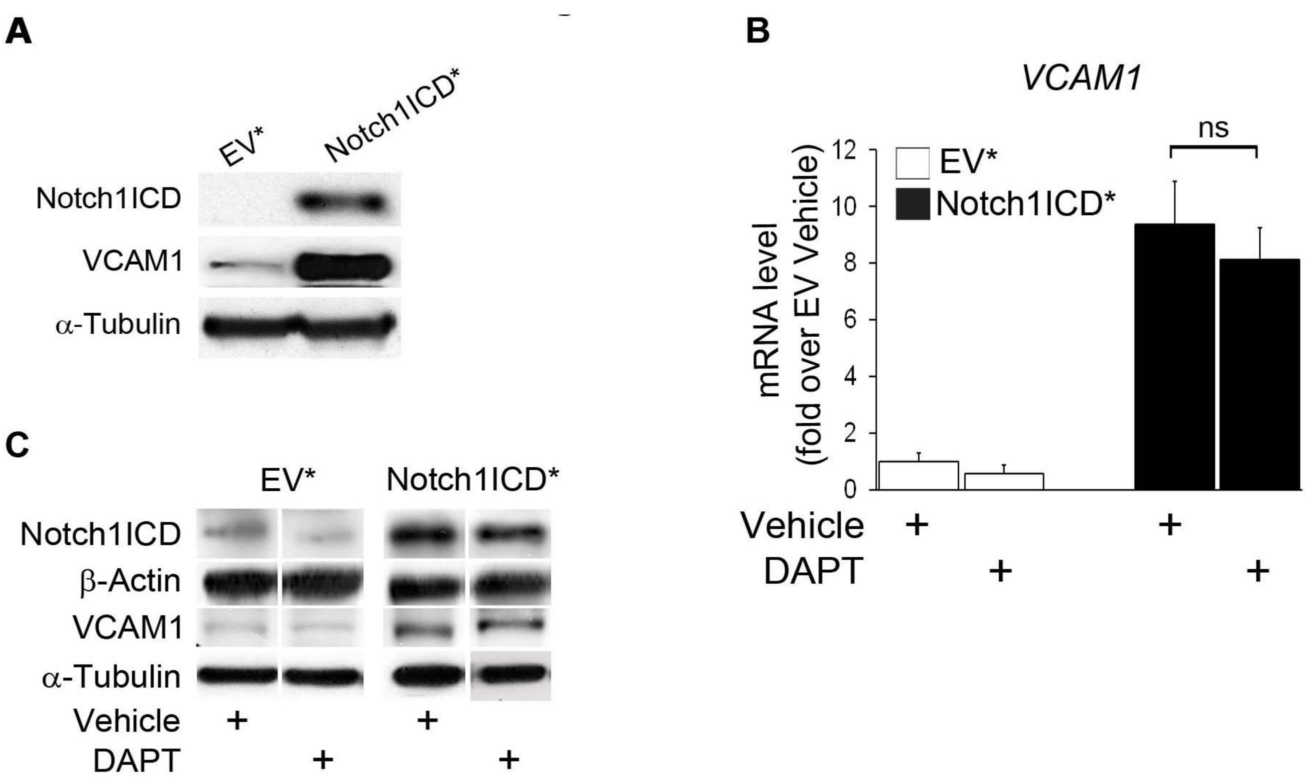

Supplementary Figure 4: Pharmacological inhibition of Notch signaling does not overcome VCAM1 upregulation induced by Notch1ICD forced expression. HUVECs were infected with either a retroviral vector co-expressing a flag-tagged murine Notch1ICD (Notch1ICD*) and the enhanced *Green Fluorescent Protein* (eGFP) or an eGFP vector (EV*) as control. (**A**) After 48 h of infection cells were analyzed by Western blot for the overexpression of Notch1ICD with an antibody that recognizes both the murine and human Notch1ICD forms cleaved at Val1744 (see Methods) and VCAM1. α-Tubulin is shown as loading control. (**B**) Forty-eight hours post-infection, confluent HUVECs were treated for 16 h with either the γ-secretase inhibitor DAPT (10μM) or the Vehicle (DMSO). mRNA expression levels of *VCAM1* were quantified by the $2^{(-\Delta\Delta Ct)}$ method (see Methods section) after normalization to *β-Actin* and expressed as fold-change over those of EV*-infected cells treated with Vehicle (1 arbitrary unit). Mean±SD. The experiments were performed in duplicates and independently repeated at least 2 times. (**C**) Representative Western blot showing levels of (i)

Notch1ICD using an antibody that recognizes both the murine and human Notch1ICD forms cleaved at Val1744 and (ii) VCAM1 in HUVECs treated as in (**B**). α-Tubulin and β-Actin were shown as loading controls.
